# From surgical debridement to laser-guided bone repair: An integrative approach to mandibular osteomyelitis

**DOI:** 10.1016/j.radcr.2026.05.024

**Published:** 2026-06-06

**Authors:** Gustavo Q. Câmara, Bianca Pulino, Bruno N. Prado, Maitê Camargo, Geraldo Prestes de Camargo Filho, Raphael Capelli Guerra

**Affiliations:** a Hospital Sírio-Libanês, Instituto de Ensino e Pesquisa, São Paulo, São Paulo, Brazil; b Oral and Maxillofacial Surgery Department, Faculdade Israelita de Ciências da Saúde Albert Einstein, São Paulo, Brazil; c Oral and Maxillofacial Surgery Department, Hospital Israelita Albert Einstein, São Paulo, Brazil; d College of Dentistry, Nova Southeastern University, Fort Lauderdale, Fl, USA

**Keywords:** Photodynamic therapy, Osteomyelitis, Low-intensity laser therapy, Methylene blue, Mandible

## Abstract

Chronic mandibular osteomyelitis is a challenging condition due to impaired vascularization, necrotic bone, and persistent infection, often requiring combined therapeutic approaches. We report the case of a 49-year-old male presenting with severe mandibular pain, purulent discharge, and radiographic evidence of bone sequestration and pathological fracture following tooth extraction. The patient was treated conservatively with surgical debridement associated with antimicrobial photodynamic therapy using 0.01% methylene blue activated by a 660 nm laser, followed by adjunctive low-level infrared laser therapy for photobiomodulation. Clinical and radiographic follow-up demonstrated resolution of infection, reduction of pain and inflammation, and progressive bone repair without adverse effects. This case highlights that the combination of photodynamic therapy and photobiomodulation may represent a safe and effective adjunctive approach in the management of chronic mandibular osteomyelitis, particularly in patients seeking less invasive alternatives to radical surgical procedures.

## Introduction

Chronic mandibular osteomyelitis remains a therapeutic challenge due to compromised local vascularization, the presence of necrotic bone, and the formation of bacterial biofilms, which significantly reduce the efficacy of systemic antibiotic therapy. The condition is commonly associated with odontogenic infections, trauma or surgical procedures and may persist despite appropriate conventional treatment, including surgical debridement and prolonged use of antibiotics [1,2].

Antimicrobial photodynamic therapy (aPDT) has emerged as a promising adjuvant modality for infection control. This approach combines a photosensitising agent, visible light of a specific wavelength, and oxygen to generate reactive oxygen species capable of destroying microbial cells without inducing bacterial resistance [3,4]. Methylene blue (MB) is one of the most widely used photosensitizers due to its affinity for microbial membranes and its favorable photochemical properties when activated by red light (∼660 nm) [5].

In addition to its antimicrobial action, low-level laser therapy (LLLT), particularly in the infrared spectrum, has demonstrated beneficial effects in modulating inflammation, angiogenesis and bone tissue repair [6]. Therefore, the combined use of aPDT and LLLT may improve clinical outcomes in chronic bone infections.

This article reports a clinical case of chronic mandibular osteomyelitis treated with surgical debridement combined with aPDT using MB and red lasers, followed by infrared laser therapy for biomodulation.

## Case report

A 49-year-old male presented to the accident and emergency department of Santa Teresa Hospital with an acute infection and severe pain localized in the right mandibular region. The patient reported a 4-week history of persistent symptoms following tooth extraction, with a nonhealing postextraction socket and continuous purulent discharge. Initial management prior to hospital admission included oral antibiotic therapy with amoxicillin–clavulanate (Clavulin BD 875 mg, every 12 hours for 14 days), without clinical improvement.

The patient’s medical history was significant for type 2 diabetes mellitus and active smoking habit. He denied any previous surgical history and reported no relevant family history of systemic diseases.

Clinical examination revealed pronounced trismus, local oedema, tenderness on palpation, and active purulent exudate from the affected alveolus. Laboratory tests demonstrated elevated inflammatory markers, with a leukocyte count of 11,300 cells/µL (reference range: 4000-10,000 cells/µL) and a C-reactive protein (CRP) level of 8.9 mg/dL (reference value: <0.5 mg/dL), consistent with an active infectious process and significantly higher than normal values reported in the literature.

Computed tomography (CT) of the postextraction alveolar region of tooth no. 47 revealed irregular and poorly defined areas of bone sclerosis, disruption of the cortical plates, particularly at the lingual mandibular cortical margin, and evidence of pathological fracture in this region (Fig. 1). Additionally, bone sequestra were identified as isolated radiopaque fragments within a radiolucent area, consistent with avascular necrotic bone.

**Fig. 1:**
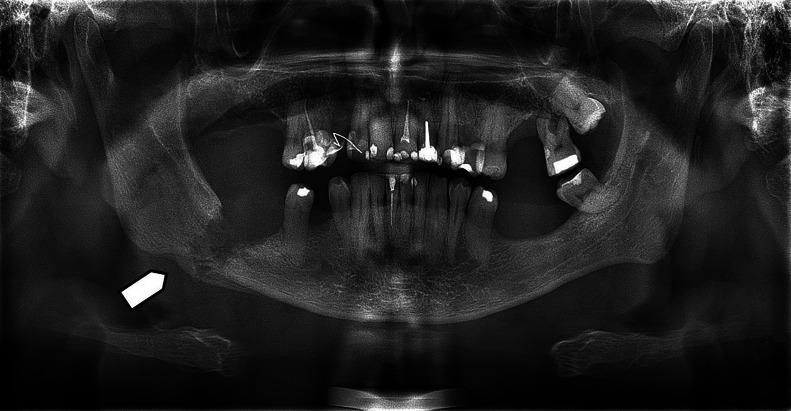
Initial computed tomography scan demonstrating irregular intraosseous radiolucent areas associated with peripheral sclerosis and bone sequestration in the mandibular region, consistent with chronic osteomyelitis, with white arrows indicating the areas of peripheral sclerosis and bone sequestration.

Given the severity of the condition, surgical treatment consisting of en bloc mandibular resection with adequate safety margins was initially proposed. However, the patient declined this approach and opted for a less invasive treatment strategy.

A conservative surgical approach was therefore performed under local anesthesia, consisting of curettage of the affected alveolar region using a spherical bur, removal of bone sequestra, debridement of adjacent infected tissues, and copious irrigation with sterile saline solution prior to the initiation of antimicrobial photodynamic therapy (aPDT).

Subsequently, aPDT was performed using 0.01% methylene blue activated by a 660 nm low-level laser, followed by adjunctive low-level infrared laser therapy for photobiomodulation. Multiple treatment sessions were carried out, with monthly radiographic follow-up over a 6-month outpatient period.

### Photodynamic therapy protocol

After irrigating the surgical cavity with sterile saline solution and gently drying it, a 0.01% MB solution was applied to the affected area using a blunt cannula, with a total volume of approximately 0.5 mL. A preirradiation time of 5 minutes was used to allow adequate penetration of the photosensitiser.

The irradiation was performed using a low-intensity red laser (660 nm, continuous wave, 100 mW). The energy applied ranged from 6 J per spot, with an exposure time of 60 seconds per spot, covering the entire affected area and its margins. The sessions were carried out once a week for 4 weeks.

### Adjuvant infrared laser therapy

Infrared laser therapy (808 nm, 100 mW) was applied in contact mode to the mandibular region to promote tissue repair and modulate inflammation. Energy doses of 8 J per point were administered at 8 points spaced approximately 1 cm apart. Sessions were conducted twice a week for 4 weeks, totaling 8 sessions on alternate days with the application of red laser.

### Clinical progress and monitoring

Patients were clinically re-evaluated during each session. A progressive reduction in pain was observed during the first 2 weeks of treatment. After the second aPDT session, purulent drainage resolved completely, accompanied by a significant reduction in oedema and bone tenderness. No clinical signs of infection progression were detected during follow-up.

Radiographic assessment was performed using panoramic radiographs at monthly intervals following the start of the treatment protocol, over a period of 6 months. Imaging studies demonstrated a progressive reduction in intraosseous radiolucent areas, associated with peripheral bone formation in previously affected regions (Fig. 2, Fig. 3). No new areas of bone rarefaction or additional sequestration were observed, indicating stabilisation of the infectious process and favourable progression of bone repair.

**Fig. 2:**
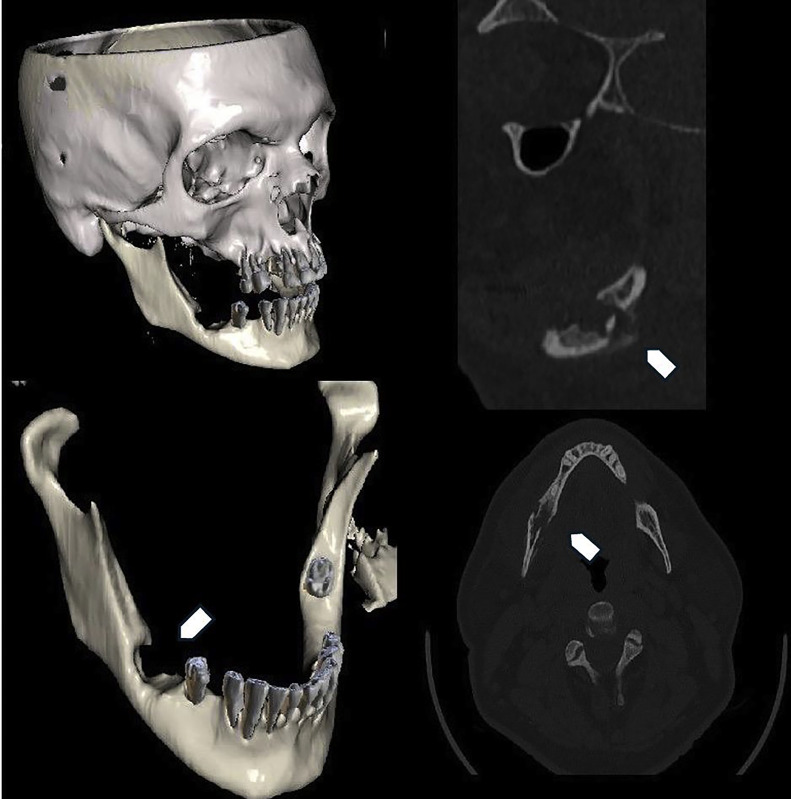
Panoramic radiograph obtained after 8 weeks of treatment, demonstrating a reduction in the intraosseous radiolucent areas and signs of bone remodeling in the mandibular region, with a white arrow indicating the area of bone remodeling.

**Fig. 3:**
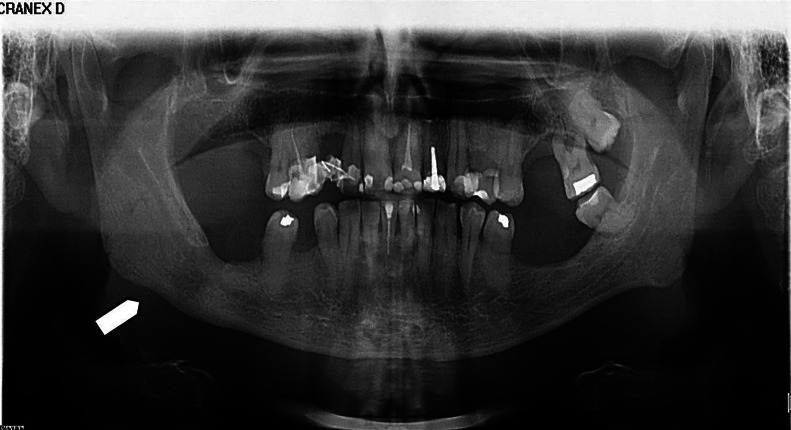
Panoramic radiograph obtained at the 6-month follow-up after completion of treatment, demonstrating increased bone remodeling and increased radiopacity in the previously affected area, with no evidence of recurrent osteolytic lesions, with white arrows indicating the areas of bone remodeling and increased radiopacity.

No adverse effects related to the photosensitizer or laser application were reported.

## Discussion

Chronic mandibular osteomyelitis remains a complex condition to manage due to the combined effects of necrotic bone, reduced vascularisation and the persistence of polymicrobial biofilms, all of which limit the efficacy of systemic antibiotic therapy [1,2]. Conventional treatment is based on surgical debridement associated with prolonged antibiotic use; however, recurrence and incomplete resolution are not uncommon, particularly in advanced or refractory cases [3,4].

In the present case, a conservative multimodal strategy combining surgical debridement, antimicrobial photodynamic therapy (aPDT) and adjunctive photobiomodulation resulted in favourable clinical and radiographic outcomes. The uniqueness of this report lies in the successful management of a case with radiographic evidence of bone sequestration and pathological fracture without the need for segmental mandibular resection, which is often recommended in similar scenarios. This finding suggests that, in selected patients, less invasive approaches may be effective while reducing surgical morbidity.

The use of aPDT as an adjunct in osteomyelitis is supported by its well-established antimicrobial mechanism, which involves the activation of a photosensitiser by light in the presence of oxygen, leading to the production of reactive oxygen species capable of inducing irreversible microbial damage [5,7]. Importantly, this mechanism is nonspecific and does not promote bacterial resistance, which is particularly advantageous in chronic infections characterised by biofilm formation and polymicrobial flora [6]. Studies by Garcez et al. [8,9] demonstrated significant microbial reduction when aPDT was used as an adjunct in endodontic infections, reinforcing its applicability in contaminated and necrotic environments. Although most available evidence is derived from dental and soft tissue infections, the present case extends these findings to infected mandibular bone, supporting the potential role of aPDT in osteomyelitis.

Another relevant aspect is the ability of aPDT to act locally in poorly vascularised tissues, where systemic antibiotics may have limited penetration. This is particularly important in chronic mandibular osteomyelitis, where sclerosis and ischaemia contribute to disease persistence. The rapid resolution of purulent drainage observed after the initial treatment sessions in this case is consistent with the antimicrobial efficacy reported in previous studies [8,10].

In addition to infection control, adjunctive low-level laser therapy (LLLT) was used to promote photobiomodulation. Experimental and clinical studies have demonstrated that LLLT can modulate inflammatory mediators, stimulate angiogenesis and enhance osteoblastic activity, thereby accelerating bone repair [[11], [12], [13]]. In the context of osteomyelitis, where chronic inflammation and impaired bone remodelling coexist, these effects may play a critical role in achieving tissue regeneration. The progressive radiographic evidence of bone formation observed over the 6-month follow-up in this case supports the potential contribution of LLLT to the healing process.

From a clinical perspective, this case highlights several important implications. First, aPDT may represent an effective adjunctive modality for controlling infection in hypovascular bone, particularly in cases where conventional antibiotic therapy is insufficient. Second, the integration of photobiomodulation may enhance tissue repair and improve overall treatment outcomes. Third, a conservative and tissue-preserving approach may be considered in selected patients, especially when radical surgical procedures are contraindicated or declined.

Despite these encouraging findings, this report is limited by its single-case design and the relatively short follow-up period. The heterogeneity of osteomyelitis in terms of microbial profile, disease extent and host response underscores the need for individualised treatment planning. Furthermore, there is currently no consensus regarding optimal aPDT parameters, including photosensitiser concentration, wavelength, energy density and treatment frequency [6,14].

Future prospective and randomised clinical studies are required to validate the role of aPDT and photobiomodulation in chronic mandibular osteomyelitis and to establish evidence-based protocols for their incorporation into routine clinical practice.

## Conclusion

This case report demonstrates the safety and clinical efficacy of antimicrobial photodynamic therapy (aPDT) using 0.01% MB, activated by a 660 nm diode laser, as an adjunct in the treatment of chronic mandibular osteomyelitis. The combination of aPDT with low-intensity infrared laser therapy for photobiomodulation resulted in significant clinical improvements, including progressive reduction in pain, complete resolution of purulent discharge, reduction in local oedema, and recovery of mandibular function. Radiographic follow-up confirmed a reduction in intraosseous radiolucent areas and the formation of new bone at the periphery, demonstrating adequate control of the infection and tissue repair.

These findings highlight that aPDT is a valuable adjunctive strategy, particularly in chronic or refractory cases where conventional approaches may be limited by impaired vascularization, biofilm formation, or reduced antibiotic penetration. Red light-activated MB provided localized antimicrobial action, whilst infrared photobiomodulation may have contributed to the modulation of inflammation and the stimulation of osteogenic activity.

Although encouraging, treatment outcomes may vary according to the extent of the disease, chronicity, and patient-related factors, reinforcing the need for individualized protocols [15]. Larger prospective randomized clinical trials are required to validate these results and establish standardized, evidence-based protocols for routine clinical practice worldwide Table 1.

**Table 1:** Laser parameters used for antimicrobial photodynamic therapy and photobiomodulation.

Therapeutic purpose	Laser type	Wavelength (nm)	Output power (mW)	Mode	Energy per spot (J)	Irradiation time (s)	Energy density (J/cm²)	Application technique	Number of points	Frequency of sessions
Antimicrobial photodynamic therapy (aPDT)	Low-power diode laser	660	100	Continuous wave	6	60	60	By contact, intracavitary or transmucosal	10	Once a wk (4 sessions)
Photobiomodulation (LLLT)	Low-intensity diode laser	808	100	Continuous wave	8	80	40	Contact, transmucosal	8	Twice a wk (4 wks)

• For antimicrobial photodynamic therapy, 0.01% MB was used as a photosensitiser, with a preirradiation time of 5 minutes prior to laser activation.

• The irradiation time was calculated using the formula:

$t\left(s\right)=\frac{Energy(J)}{Power(W)}$

• The number of irradiation points was adjusted according to the extent of the osteomyelitic lesions.

• Laser irradiation was performed using the contact technique to ensure complete coverage of the affected area and the margins of the lesion.

## Patient consent

A written informed consent was obtained from the patient for the publication of this case report.
